# Supplementary material for: Spectroscopic evidence of superconductivity pairing at 83 K in single-layer FeSe/SrTiO3 films
Source: Nat Commun. 2021 May 14;12:2840. doi: 10.1038/s41467-021-23106-y (PMC8121788; doi:10.1038/s41467-021-23106-y)
Supplement: Supplementary file 1 — Supplementary Information [file 41467_2021_23106_MOESM1_ESM.pdf]

Supplementary Materials for

Spectroscopic Evidence of Superconductivity Pairing at 83 K in Single-Layer $FeSe/SrTiO_3$ Films

Yu Xu[1,2♯], Hongtao Rong[1,2♯], Qingyan Wang[1,2*,♯], Dingsong Wu[1,2♯], Yong Hu[1,2], Yongqing Cai[1,2], Qiang Gao[1,2], Hongtao Yan[1,2], Cong Li[1,2], Chaohui Yin[1,2], Hao Chen[1,2], Jianwei Huang[1], Zhihai Zhu[1,2], Yuan Huang[1,2], Guodong Liu[1,2,3], Zuyan Xu[4], Lin Zhao[1,2,3*] and X. J. Zhou[1,2,3,5*]

[1]*National Lab for Superconductivity,*

Beijing National Laboratory for Condensed Matter Physics,

Institute of Physics, Chinese Academy of Sciences, Beijing 100190, China

[2]*University of Chinese Academy of Sciences, Beijing 100049, China*

[3]*Songshan Lake Materials Laboratory, Dongguan 523808, China*

[4]*Technical Institute of Physics and Chemistry,*

Chinese Academy of Sciences, Beijing 100190, China

[5]*Beijing Academy of Quantum Information Sciences, Beijing 100193, China*

[♯]*These authors contribute equally to the present work.*

[*]*Corresponding author: qingyanwang@iphy.ac.cn,*

lzhao@iphy.ac.cn and XJZhou@iphy.ac.cn

Supplementary Note 1. Band splitting resolved in band structure measured at 83 K along momentum Cut 1 in single-layer FeSe/STO films

Supplementary Fig. 1a shows a Fermi surface mapping of the single-layer FeSe/STO films. Supplementary Fig. 1b shows the band structure measured at 83 K along the momentum Cut 1 with its location marked by a red line in Supplementary Fig. 1a. Supplementary Fig. 1c shows the corresponding second derivative image from Supplementary Fig. 1b. The band splitting can be seen from Supplementary Fig. 1c. Supplementary Fig. 1d shows MDCs from Supplementary Fig. 1b at two energies: one is 5 meV above the Fermi level while the other is 16 meV below the Fermi level. Two peaks can be resolved from these MDC peaks. This makes it possible to extract quantitatively the band structure at 83 K, as shown in Fig. 1f. Supplementary Fig. 1e shows the band structure measured at 41 K along the momentum Cut 1 in which the Bogoliubov back-bending band above Fermi level can be observed. Supplementary Fig. 1f shows the EDC along the black dashed line at k_F in Supplementary Fig. 1e which is consistent with the particle-hole symmetry.

Supplementary Note 2. The measured and extracted band structures in normal and superconducting states along momentum Cut 1 in single-layer FeSe/STO films

We extract the band structures from the raw data measured at 83 K (Supplementary Fig. 2a) and 20 K (Supplementary Fig. 2d) along the momentum Cut 1 (Supplementary Fig. 1a) as shown by the solid lines in Supplementary Fig. 2a and Supplementary Fig. 2d, respectively. Taking the normal state band structures (superconducting state band structures) and the superconducting gap size for the two bands, we can extract the band structure in the superconducting state (normal state) by the BCS formula $E_k = -[(\xi_k)^2 + (\triangle_k)^2]^{1/2}$ where ξ_k represents the normal state band structure, E_k represents the superconducting band structure and $\triangle_k$ is the superconducting gap size. For example, the dashed lines in Supplementary Fig. 2b represent the extracted superconducting band structures by the BCS formula taking the normal state band structures (Supplementary Fig. 2a), and the gap size of $\triangle_1 \sim 9meV$ for the inner band and $\triangle_2 \sim 13meV$ for the outer band. The extracted band structures agree well with the measured ones in Supplementary Fig. 2d. Furthermore, we can extract the normal state band structure extending to ~80 meV above the Fermi level by the BCS formula due to the very strong Bogoliubov back-bending bands in the superconducting state (Supple-

mentary Fig. 2d). The form of the two lines in Supplementary Fig. 2d are as follow: $E_{blue} = -((8.088*(k+1.414)^6 - 4.6231*(k+1.414)^4 + 1.228*(k+1.414)^2 - 0.0554)^2 + 0.009*0.009)^{0.5}$, $E_{black} = -((1.1451*(k+1.414)^6 - 1.3703*(k+1.414)^4 + 0.74814*(k+1.414)^2 - 0.054573)^2 + 0.013*0.013)^{0.5}$.

Supplementary Note 3. The measured and extracted band structures in normal and superconducting states along momentum Cut 2 in single-layer FeSe/STO films

Supplementary Fig. 3 shows the measured and extracted band structures following the same procedure as used in Supplementary Fig. 2. In normal state (Supplementary Fig. 3a), the band structure from FS 2 (Fig. 1a) is very weak, so we can only extract the band structure from FS 1 (Fig. 1a). On the other hand, in superconducting state, we can only resolve the band structure from FS 2. The change of the spectral weight of the two bands with temperature is unusual which is probably due to combined effects of light polarization, their orbital character and photoemission matrix element effects.

Supplementary Note 4. Detailed analysis of the back-bending band

Supplementary Fig. 4a shows the band structure measured along the momentum Cut 2 at 20 K. The image is divided by the corresponding Fermi distribution function. The band below E_F marked by blue solid line is from superconductivity-induced Bogoliubov back-bending band. There is a dramatic spectral intensity change with energy, as shown by EDCs (energy distribution curves) and MDCs (momentum distribution curves) for the selected 5 typical points (A, B, C, D and E in Supplementary Fig. 4a) on the back-bending band. From the photoemission spectra (EDCs) in Supplementary Fig. 4c, it is clear that the spectral intensity drops rapidly with energy from A, B to C points. It looks like that there is little intensity change with binding energy for the Bogoliubov back-bending bands from C to D to E. This is due to the fact that the back-bending bands sit on a shoulder of a big background that comes from the central band, as shown by the MDCs at three different binding energies in Supplementary Fig. 4d. The net spectral weight of the back-bending band still decreases with increasing binding energy, as shown in Supplementary Fig. 4e. Overall, from A to E point, there is a dramatic spectral weight decrease with increasing binding energy that is consistent with the behavior expected with the usual Bogoliubov

back-bending band.

Furthermore, the back-bending bands we observed are consistent with the behaviors expected from the BCS theory. We have carried out similar analysis of the coherence factors and spectral weight distribution of the back-bending bands, as done before in[1]. According to the BCS theory, the coherence factors can be extracted from the energy bands in the normal state and in the superconducting state in the following way: $|u_k|^2 = 1 - |v_k|^2 = 1/2(1 + \xi_k/E_k)$ where ξ_k and E_k are the energy of the normal state band and Bogoliubov back-bending band, respectively. Supplementary Fig. 4b shows the extracted $|u_k|^2$and $|v_k|^2$ obtained this way.

We also make a quantitative analysis on the intensity change of the back-bending band. We plot EDCs in Supplementary Fig. 4c and MDCs in Supplementary Fig. 4d,e to extract the spectral weight of the back-bending band at 5 typical points on the back-bending band. After proper renormalization of the spectral intensity, we find that the intensity change of the back-bending band as a function of the binding energy follows closely with the obtained coherence factor $|u_k|^2$. Therefore, the back-bending bands we observed are consistent with all the behaviors of the Bogoliubov back-bending bands, expected from the BCS theory. There is no doubt that the back-bending bands we observed are from superconductivity-induced Bogoliubov back-bending bands.

Supplementary Note 5. Temperature dependence of photoemission spectra along momentum Cut 1 in single-layer FeSe/STO films

We can clearly observe two parabolic bands (Supplementary Fig. 5a) that are gapped with different gap size. Photoemission spectra (EDCs) on the four Fermi momenta of the two bands can be obtained at the same time. Supplementary Fig. 5b and Supplementary Fig. 5c show symmetrized EDCs at the Fermi momenta from the inner band and outer band, respectively. The EDCs extracted from the inner parabolic band show two clear peaks below E_F at low temperature (Supplementary Fig. 5b); the peak at high binding energy comes from the outer band. On the other hand, the EDCs extracted from the outer parabolic band show only one peak below E_F at low temperature (Supplementary Fig. 5c).

Supplementary Note 6. Temperature dependence of the energy gap and the associated spectral weight along a Fermi surface in Bi2212

In order to examine on the analysis methods, and to compare with the results of single-layer FeSe/STO films, we follow the same procedure used in Fig. 2 to analyze experimental data from Bi2212 with a superconducting critical temperature T_c=91 K[2]. In Bi2212, one main band is observed (Supplementary Fig. 6b) along a momentum cut shown in Supplementary Fig. 6a. Supplementary Fig. 6c shows the original EDCs measured at the Fermi momentum at different temperatures, and the corresponding symmetrized EDCs are shown in Supplementary Fig. 6d. The energy gap obtained from the symmetrized EDCs in Supplementary Fig. 6d following the usual procedure[3] is plotted in Supplementary Fig. 6g (black circles). Following the procedure in Fig. 2, we also get the EDCs with its Fermi distribution function directly removed as shown in Supplementary Fig. 6e, and furthermore, get normalized EDCs from dividing each EDC in Supplementary Fig. 6e with the one at 180 K, as shown in Supplementary Fig. 6f. The energy gap obtained from Supplementary Fig. 6f by picking up the peak position of the normalized EDCs is also plotted in Supplementary Fig. 6g (red squares). The energy gap obtained from these two methods are consistent. In Supplementary Fig. 6h, we take three spectral intensities: the height of the peak below the Fermi level, the height of the dip at the Fermi level and the difference between the peak height and the dip height (PDD). In the temperature region of 91-140 K, the peak-dip structure is visible but does not show strong temperature dependence which corresponds to the formation of pseudogap as shown in Supplementary Fig. 6g. In a strong contrast, below T_c=91 K, all the features become clear and they all exhibit a dramatic temperature dependence when the sample is in superconducting state. The electronic behaviors we observed in single-layer FeSe/STO films (Fig. 2g and Fig. 3e) are similar to that observed in Bi2212 (Supplementary Fig. 6h).

Supplementary Note 7. Temperature dependence of photoemission spectra along momentum Cut 2 in single-layer FeSe/STO films

Supplementary Fig. 7a shows the band structure of the single-layer FeSe/STO films measured along the momentum Cut 2 (Fig. 1a). A strong flat band is observed in the central region while superconductivity-induced Bogoliubov back-bending bands are observed on both sides. Supplementary Fig. 7b shows the EDCs measured at the center momentum (k_center marked by the arrow in Supplementary Fig. 7a) at different temperatures, and the corresponding symmetrized EDCs are shown in Supplementary Fig. 7c. Supplementary

Fig. 7d shows EDCs obtained from Supplementary Fig. 7b by dividing out the corresponding Fermi distribution functions. Supplementary Fig. 7e shows normalized EDCs that are obtained from Supplementary Fig. 7d by dividing the EDCs at low temperatures with the EDC at 103 K. Supplementary Fig. 7f shows the gap size extracted from the EDCs in Supplementary Fig. 7c and Supplementary Fig. 7e. The black circles show the energy gap obtained by picking the peak position of the symmetrized EDCs in Supplementary Fig. 7c. The red circles show the gap obtained by picking the peak position of the normalized EDCs in Supplementary Fig. 7e. The results are similar to those measured along the Cut 1.

Supplementary Note 8. Extraction of the spectral weight of the superconductivity-induced Bogoliubov back-bending band from the momentum Cut 2 measurements in single-layer FeSe/STO films

Supplementary Fig. 8a shows the band structure of the single-layer FeSe/STO films measured along the momentum Cut 2 (Fig. 1a). Supplementary Fig. 8b shows momentum distribution curves (MDCs) measured at different temperatures. The MDCs are obtained by integrating over an energy range of [-30, -60] meV, as marked by the two grey horizontal lines in Supplementary Fig. 8a. These MDCs are normalized to have the same peak height around the momentum $k = 1.414\pi/a$. The normalized MDCs show a good agreement with each other at the top-right region. The extra spectral weight in MDCs on the right shoulder at low temperatures comes from the Bogoliubov back-bending band. Fig. 3d and Fig. 4e are obtained by integrating the spectral weight of these MDCs over the momentum range defined by the two red dashed lines in Supplementary Fig. 8b. This gives the spectral weight change of the Bogoliubov back-bending band with temperature in the enclosed energy-momentum box BB defined by the red dashed lines in Supplementary Fig. 8a.

---

[1] H. Matsui et al., BCS-Like Bogoliubov quasiparticles in high-T_c superconductors observed by angle-resolved photoemission spectroscopy. Phys. Rev. Lett. **90**, 217002 (2003).

[2] X. Sun et al., Temperature evolution of energy gap and band structure in the superconducting and pseudogap states of $Bi_2Sr_2CaCu_2O_{8+\delta}$ superconductor revealed by laser-based angle-

resolved photoemission spectroscopy. Chin. Phys. Lett. **35**, 017401 (2018).

[3] M. R. Norman et al., Phenomenology of the low-energy spectral function in high-T_c superconductors. Phys. Rev. B **57**, R11093 (1998).

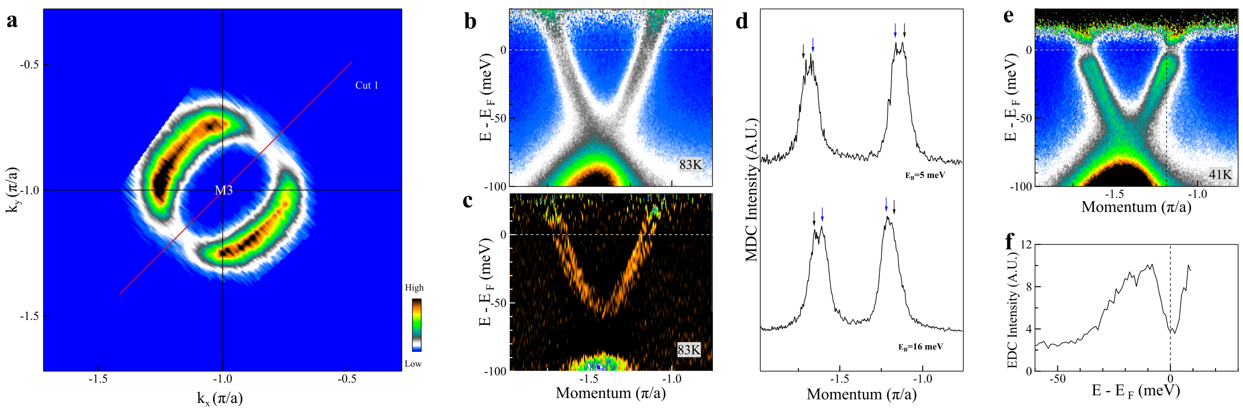

Supplementary Fig. 1. **Fermi surface mapping and band structure measured along momentum Cut 1.** (a) Fermi surface mapping near M3 (-π,-π) measured at 20 K. It is obtained by integrating the spectral weight within [-5,5] meV energy window with respect to the Fermi level (same as Fig. 1a). (b) Band structure measured along the momentum Cut 1 at 83 K. The image is obtained from dividing the original data by the corresponding Fermi distribution function. The location of the momentum Cut 1 is shown in (a). (c) Corresponding second derivative image obtained from (b) with respect to the energy. (d) Representative MDCs of the photoemission image in (b) at different binding energies. The upper MDC is at 5 meV above the Fermi level while the lower one is at 16 meV below the Fermi level. The splitting of the main bands is marked by two colored arrows. (e) Band structure measured at 41 K along the momentum Cut 1. The image is obtained from dividing the original data by the corresponding Fermi distribution function. (f) EDC along the black dashed line at k_F in (e).

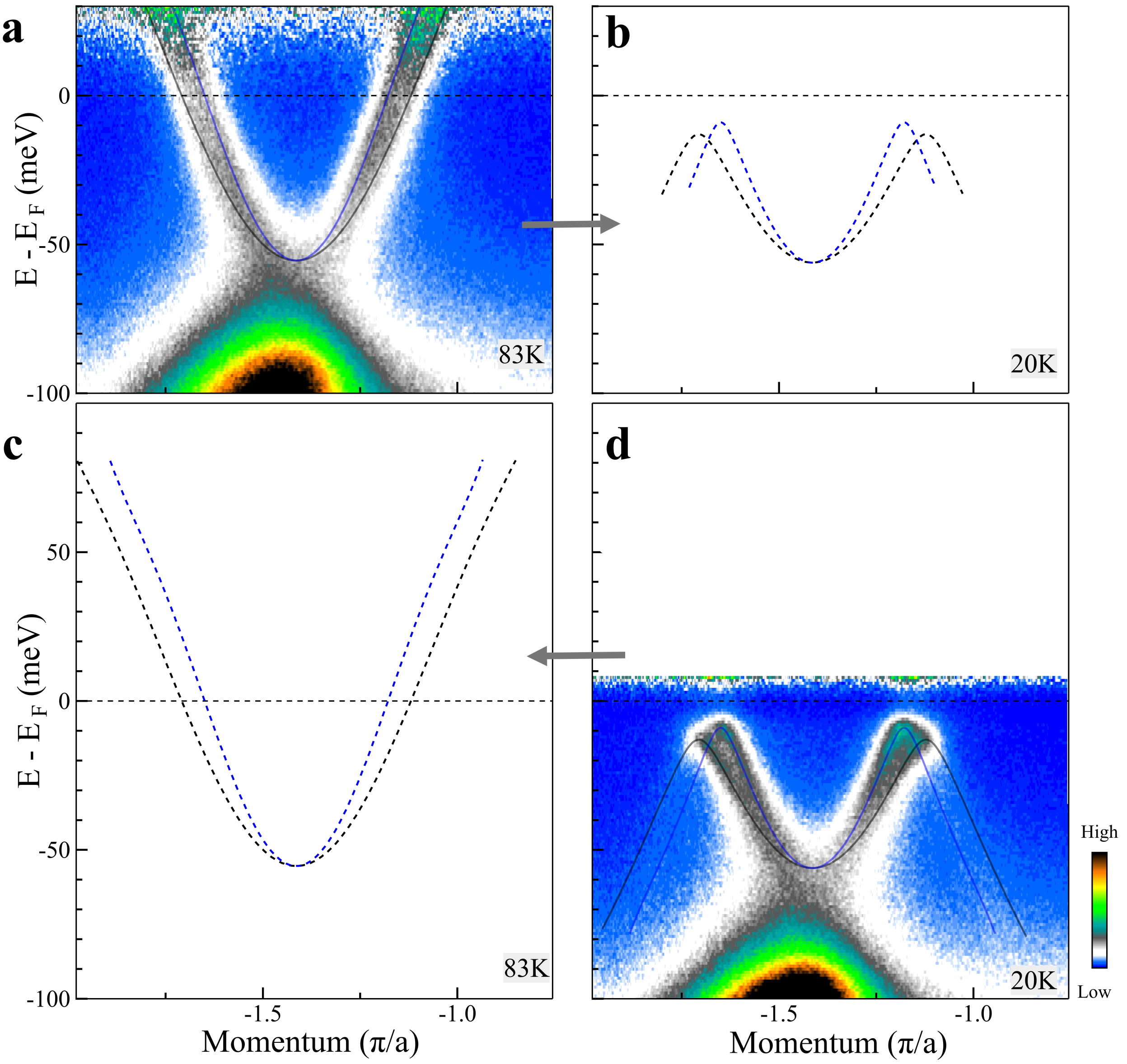

Supplementary Fig. 2. **The measured and extracted band structures along momentum Cut 1.** (a) Band structure measured along the momentum Cut 1 at 83 K. The image is divided by the corresponding Fermi distribution function. The location of the momentum Cut 1 is shown in Fig. 1a. The solid lines in the image represent extracted band structures that are same as those in Fig. 1f. (b) Extracted band structures in the superconducting state by the BCS formula taking the normal state band structures in (a), and the energy gaps of 9 meV and 13 meV for the inner and outer bands, respectively. (c) Extracted band structures in the normal state by the BCS formula taking the superconducting state band structures in (d), and the energy gaps of 9 meV and 13 meV for the inner and outer bands, respectively. (d) Band structure measured along the momentum Cut 1 at 20 K. The image is divided by the corresponding Fermi distribution function. The solid lines in the image represent extracted band structures that are same as those in Fig. 1g.

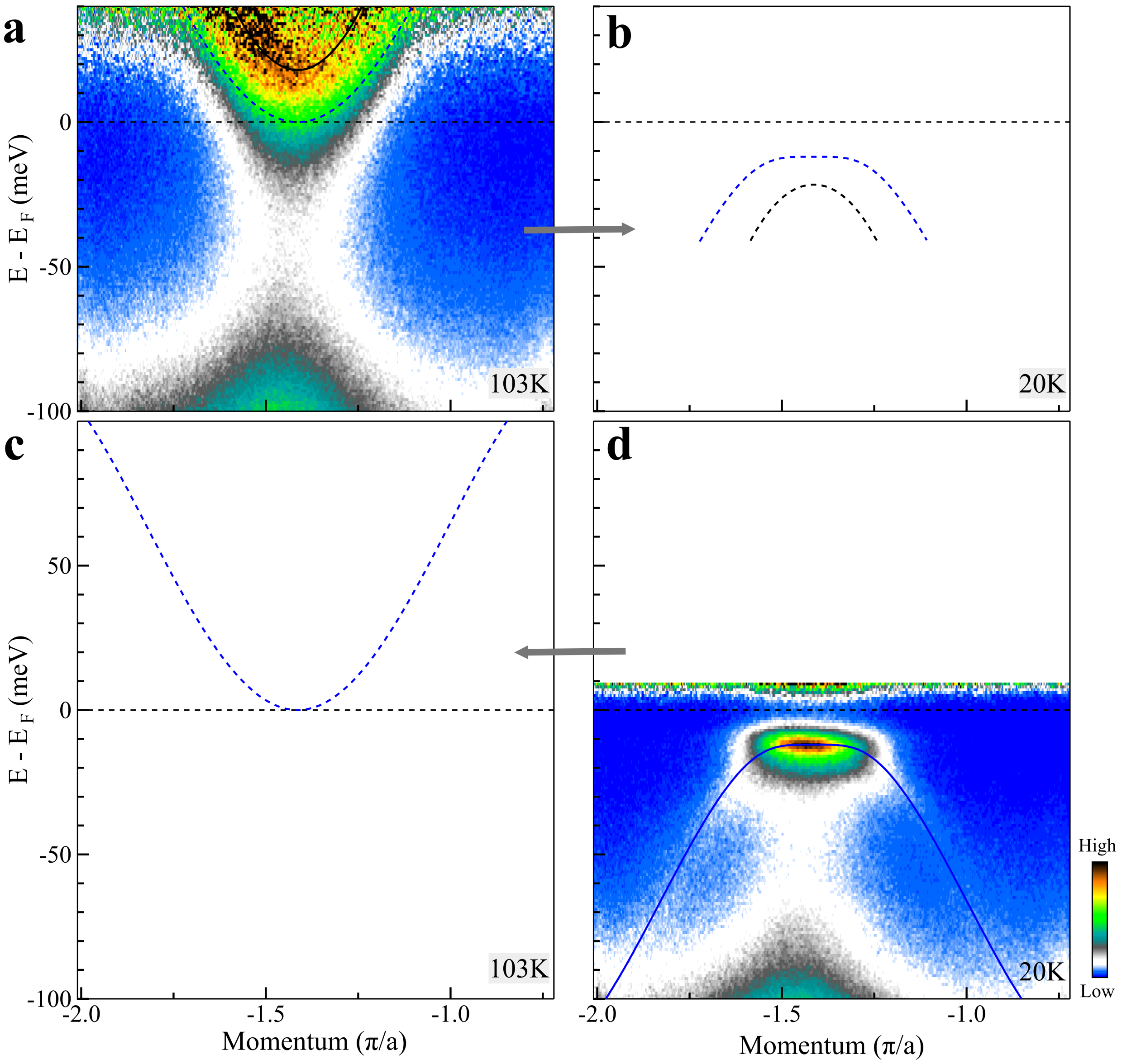

Supplementary Fig. 3. **The measured and extracted** band structures along momentum Cut 2. (a) Band structure measured along the momentum Cut 2 at 103 K. The image is divided by the corresponding Fermi distribution function. The location of the momentum Cut 2 is shown in Fig. 1a. The solid line in the image represents extracted band structure that is same as that in Fig. 1l. The band marked by dashed blue line is expected from the overall measured Fermi surface and band structure. (b) Extracted band structure in the superconducting state by the BCS formula taking the normal state band structure in (a), and an energy gap of 12 meV. (c) Extracted band structure in the normal state by the BCS formula taking the superconducting state band structure in (d), and an energy gap of 12 meV. (d) Band structure measured along the momentum Cut 2 at 20 K. The image is divided by the corresponding Fermi distribution function. The solid line in the image represents extracted band structure that is the same as that in Fig. 1m.

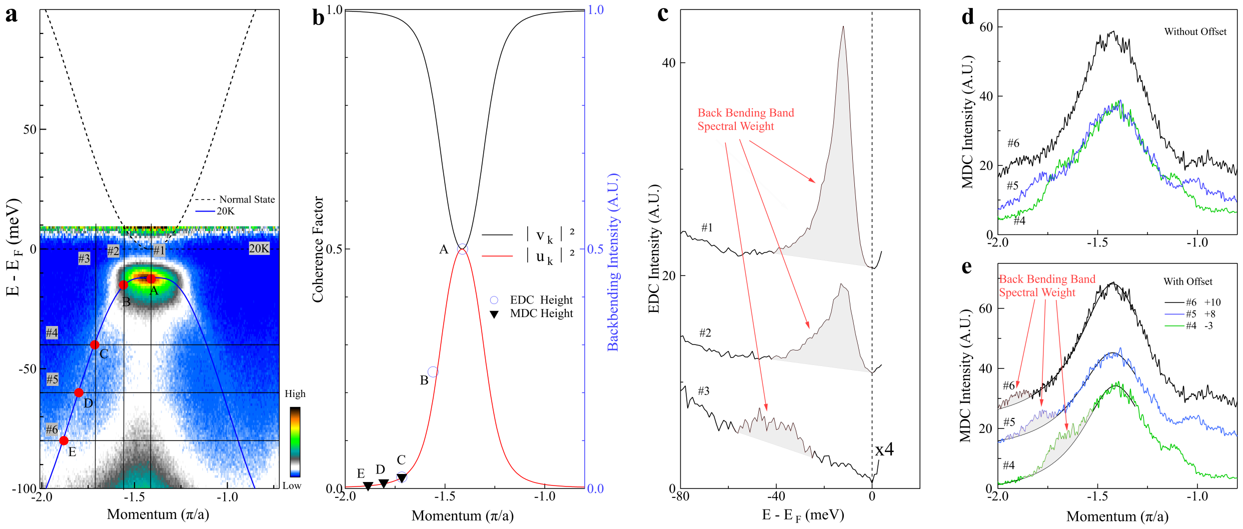

Supplementary Fig. 4. **Analysis of the Bogoliubov back-bending band.** (a) Bogoliubov back-bending band formed at 20 K for the momentum Cut 2. The black dashed line represents the initial band in the normal state while the blue solid line represents the back-bending band. (b) Coherence factors deduced from the normal state band and the back-bending band at 20 K in (a). The intensity of the back-bending band from EDC analysis (c) and MDC analysis (e) is plotted. The intensity for point A at the Fermi momentum is taken as 0.5 to normalize the EDC intensity for the point B and C. The intensity of point C is taken as the same from both the EDC and MDC analysis to normalize the spectral intensity of the D and E points. (c) EDCs along cuts ♯1, ♯2 and ♯3 as shown in (a). The spectral weight of the back-bending band is marked. (d) Momentum distribution curves (MDCs) along cuts ♯4, ♯5 and ♯6 as shown in (a). (e) Same MDCs as in (d) but are plotted with an offset for clarity. The spectral weight of the back-bending band is marked.

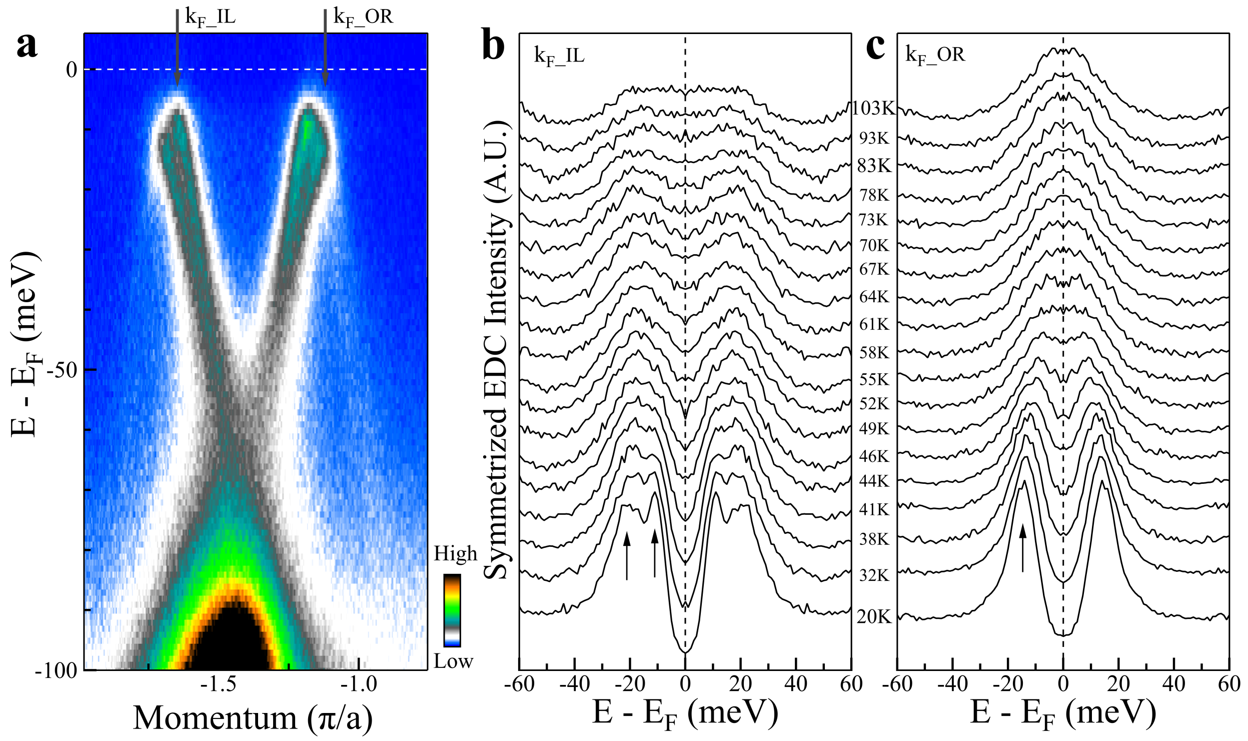

Supplementary Fig. 5. **Temperature dependence of the symmetrized EDCs along the two Fermi surface sheets.** (a) Band structure measured along the momentum Cut 1 at 20 K. The location of the momentum Cut 1 is marked in Fig. 1a that crosses both the M3($-\pi$,$-\pi$) point and the two ellipse-like Fermi surface sheets. (b) Symmetrized EDCs measured at different temperatures along the Fermi surface sheet FS 2 in Fig. 1a at the Fermi momentum k_F_IL (inner Fermi surface, left side) as marked by the arrow in (a). In this case, the symmetrized EDCs at low temperatures consist of two peaks from two bands, as marked by two arrows. The coexistence of the two peaks complicates the determination of the gap closing temperature. (c) Symmetrized EDCs measured at different temperatures along the Fermi surface sheet FS 1 in Fig. 1a at the Fermi momentum k_F_OR (outer Fermi surface, right side) as marked by the arrow in (a). In this case, the symmetrized EDCs at low temperatures consist of mainly one peak.

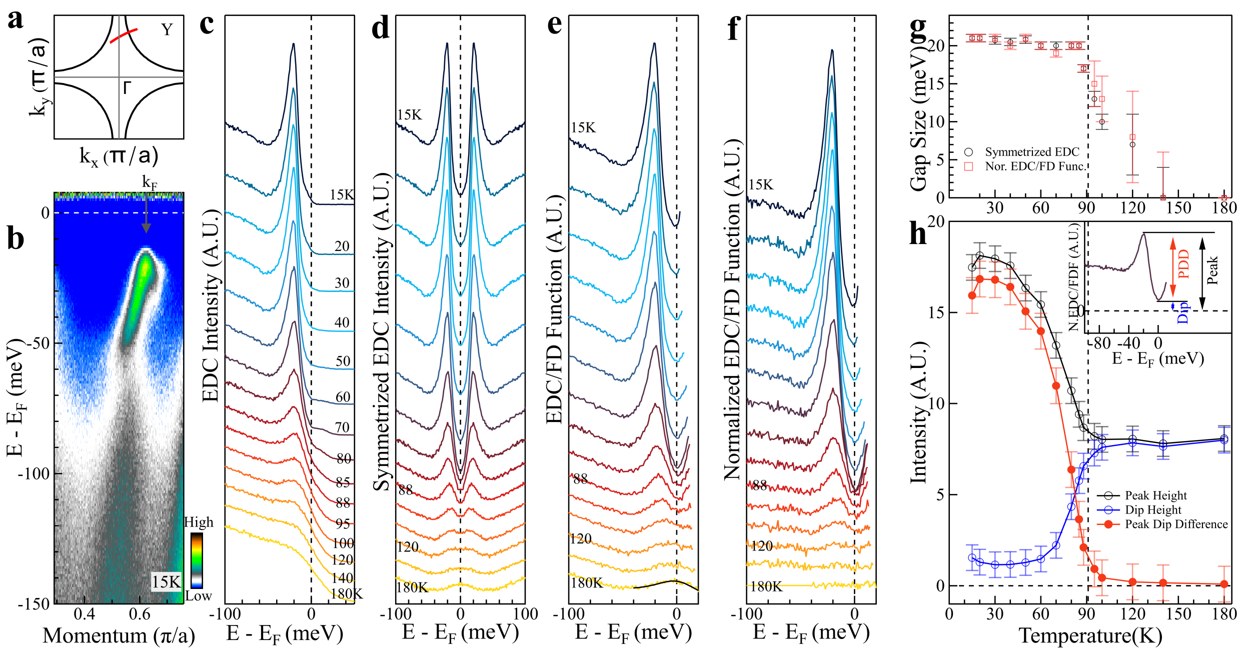

Supplementary Fig. 6. **Temperature dependence of the energy gap and the associated spectral weight along the Fermi surface in Bi2212.** (a) Schematic of the Fermi surface in Bi2212. (b) Band structure measured at 15 K along the momentum cut as shown by the red line in (a). The image is obtained by dividing the original data by the corresponding Fermi distribution function. (c) Photoemission spectra (EDCs) measured at different temperatures at the Fermi momentum k_F marked by the arrow in (b). (d) The corresponding symmetrized EDCs at different temperatures obtained from (c). (e) EDCs at different temperatures obtained from dividing the original EDCs in (c) by their corresponding Fermi distribution functions (FD Function). The solid black line at the bottom represents the fitted curve of the 180 K data by one Lorentzian. (f) EDCs at different temperatures obtained from dividing the EDCs in (e) by the fitted curve at 180 K. (g) Temperature dependence of the energy gap. The black circles show the energy gap obtained by picking the peak position of the symmetrized EDCs (d) or fitting the symmetrized EDCs with the phenomenological formula that has been commonly used for extracting the energy gap in cuprate superconductors[3]. The red squares show the gap obtained by picking the peak position of the normalized EDCs in (f). The results from these two methods are consistent. (h) Temperature dependence of the spectral weight obtained from the normalized EDCs following the same procedure as used in Fig. 2 and Fig. 3. Here we take three spectral intensities: the height of the peak below the Fermi level, the height of the dip at the Fermi level and the difference between the peak height and the dip height (PDD). The corresponding three spectral intensities as a function of temperature are shown by the empty black circles, empty blue circles and the red solid circles, respectively. Error bars reflect the uncertainty in determining the EDC peak position and spectral intensity.

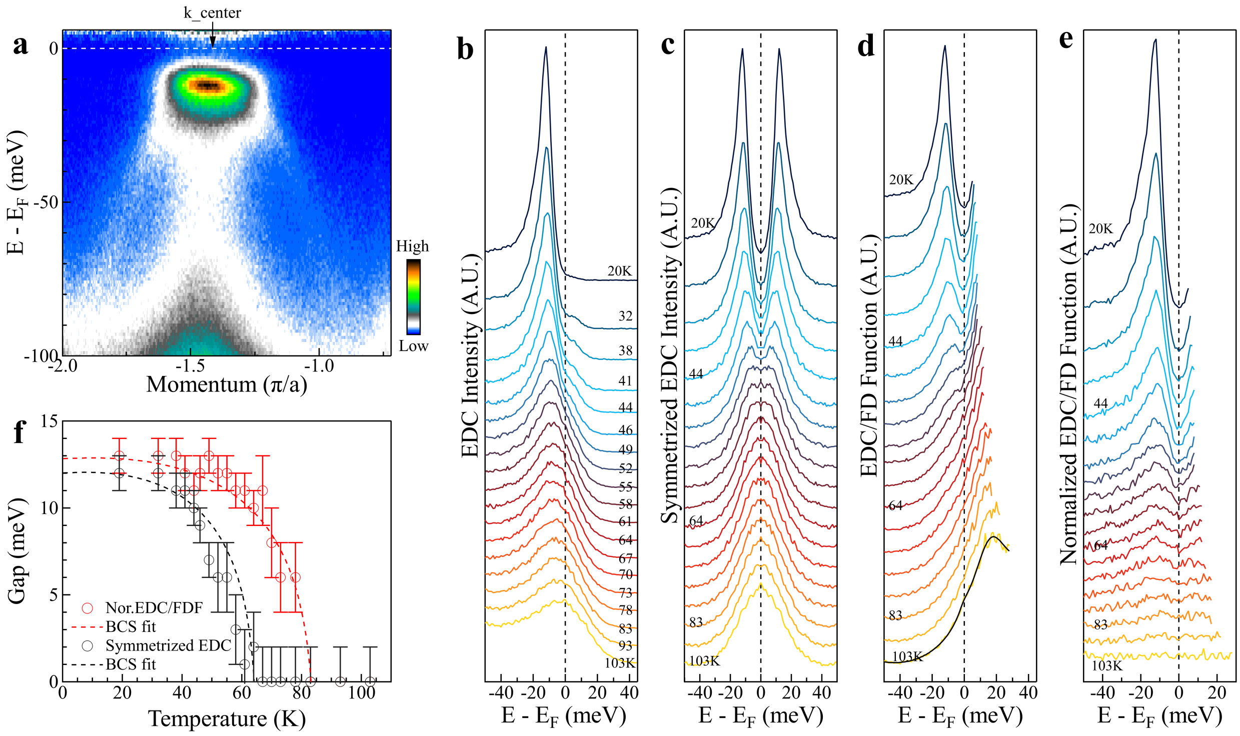

Supplementary Fig. 7. **Temperature dependence of photoemission spectra for Cut 2.** (a) Band structure measured along the momentum Cut 2 at 20 K. The location of the momentum Cut 2 is shown in Fig. 1a. The image is obtained from dividing the original data by the corresponding Fermi distribution function. (b) EDCs measured at different temperatures at the momentum k_center as marked by the arrow in (a). (c) The corresponding symmetrized EDCs at different temperatures obtained from (b). (d) EDCs at different temperatures obtained by dividing the original EDCs in (b) by their corresponding Fermi distribution functions (FD Function). The solid black line at the bottom represents the fitted curve of the 103 K data. (e) Normalized EDCs at different temperatures obtained from dividing the EDCs in (d) by the fitted curve at 103 K. (f) Temperature dependence of the energy gap. The black circles show the energy gap obtained by picking the peak position of the symmetrized EDCs in (c). The red circles show the gap obtained by picking the peak position of the normalized EDCs in (e). The dashed lines show the fitted curve of the energy gap by the BCS formula. Error bars reflect the uncertainty in determining the EDC peak position.

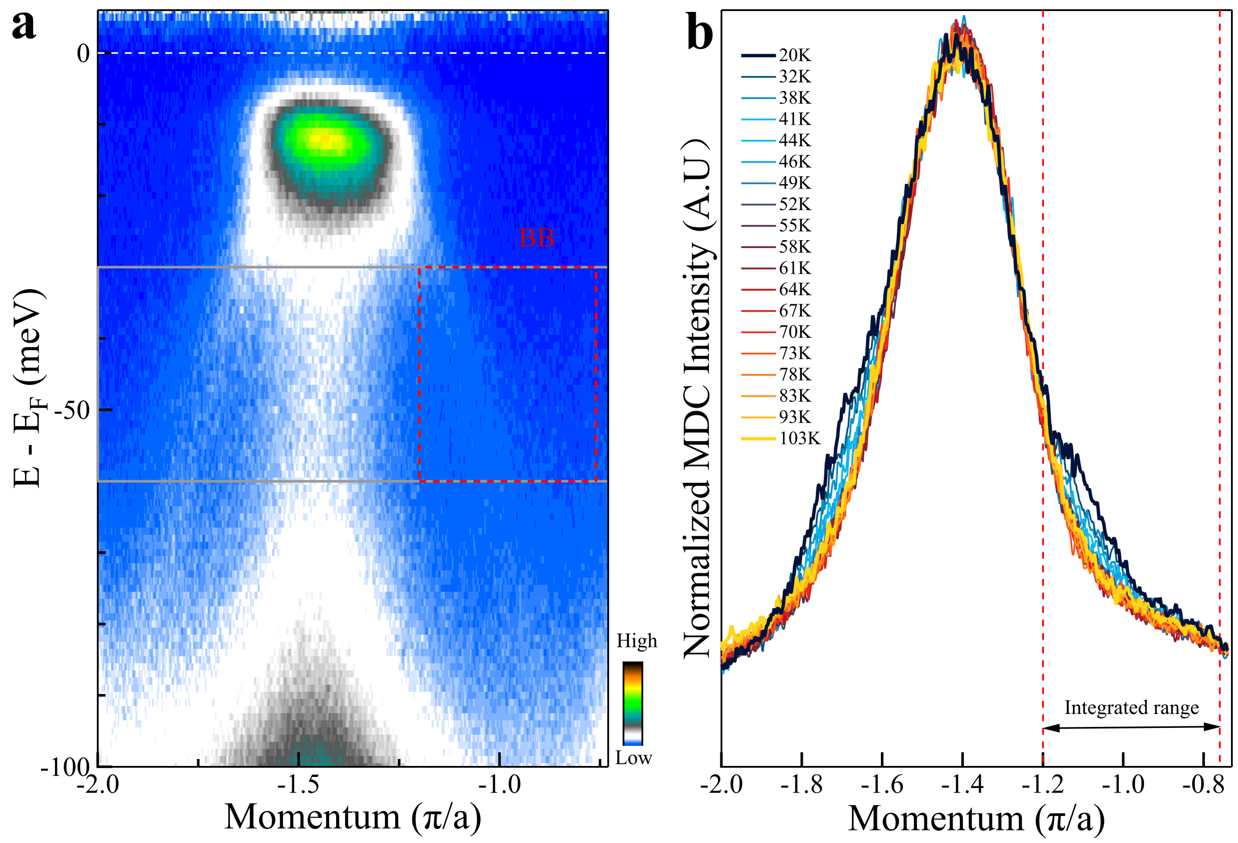

Supplementary Fig. 8. **Temperature dependence of MDCs for the momentum Cut 2 to get the spectral weight of the Bogoliubov back-bending band.** (a) Band structure measured along the momentum Cut 2 at 20 K. The location of the momentum Cut 2 is shown in Fig. 1a. The image is obtained by dividing the original data by the corresponding Fermi distribution function. (b) MDCs measured at different temperatures. The MDCs are obtained by integrating over an energy range of [-30, -60]meV, as marked by the two grey horizontal lines in (a). These MDCs at different temperatures are normalized to have the same peak height around the momentum $k = 1.414\pi/a$. The extra spectral weight in MDCs at low temperatures comes from the Bogoliubov back-bending band. Fig. 3d and Fig. 4e are obtained by integrating the spectral weight of these MDCs over the momentum range defined by the two red dashed lines. This gives the spectral weight change of the Bogoliubov back-bending band with temperature in the enclosed energy-momentum box BB defined by the red dashed lines in (a).
